# What stroke survivors say about living with upper limb spasticity and how they manage it

**DOI:** 10.1111/1440-1630.70045

**Published:** 2025-09-04

**Authors:** Shannon Pike, Natasha A. Lannin, Lisa Cameron, Mithu Palit, Emma Schneider, Anne Cusick

**Affiliations:** ^1^ School of Allied Health, Human Services and Sport (Occupational Therapy) La Trobe University Melbourne Victoria Australia; ^2^ Wagga Wagga Ambulatory Rehabilitation Service Murrumbidgee Local Health District Wagga Wagga New South Wales Australia; ^3^ School of Clinical Medicine, University of New South Wales Medicine and Health Rural Clinical Campus Wagga Wagga New South Wales Australia; ^4^ Department of Neurosciences Monash University Melbourne Melbourne Victoria Australia; ^5^ Rehabilitation, Aged and Community Care Alfred Health Melbourne Victoria Australia; ^6^ School of Health Sciences, Department of Allied Health Swinburne University of Technology Melbourne Victoria Australia; ^7^ Faculty of Medicine and Health The University of Sydney Sydney New South Wales Australia

**Keywords:** lived experience, qualitative research, spasticity, stroke, stroke rehabilitation, upper limb

## Abstract

**Introduction:**

Post‐stroke spasticity can cause serious impairment, activity limitation, and participation restrictions for survivors, leading to stroke‐related disability. While there are hundreds of qualitative studies regarding stroke survivor experience, the phenomenon of what it is like to have post‐stroke spasticity is not well understood.

**Methods:**

Ten community‐dwelling adults with chronic stroke and upper limb spasticity who had recently participated in an intensive upper limb rehabilitation programme were interviewed. A descriptive phenomenological approach using thematic analysis was used to identify patterns in the data to construct an understanding of the experience of what it is like to have upper limb spasticity.

**Consumer and Community Involvement:**

There was consumer review of the information and consent form; there was no further consumer and community involvement.

**Findings:**

Having upper limb spasticity was an embodied experience. Participants created strategies to manage the spasticity and keep active and engaged in valued everyday activities. Some strategies arose from rehabilitation experience, but most were perceived by survivors to be personally developed by them and unique to their everyday activities. These self‐management strategies were patterns of action in everyday life that were anchored in time and their access to and use of health and home‐care services and assistive technology. Self‐management strategies involved expecting, learning, practising, evaluating, and moving forward.

**Conclusion:**

Findings from this study indicate that stroke survivors who have upper limb spasticity engage in a daily process of adaptation and adjustment to stay active and engaged in valued everyday activities. Further, they use their experience in rehabilitation, access home, and health services as much as they can and develop unique strategies to self‐manage upper limb spasticity to reduce impairment and engage in activity. Strategies in this study provide insights for stroke survivors, families, and therapists on ways self‐management can be enacted from a survivor perspective.

## PLAIN LANGUAGE SUMMARY

Some people have tight or stiff muscles in their arm after a stroke. This is called spasticity. It can make it hard to move the arm and do everyday things like getting dressed or eating. Many people who have had a stroke live with arm spasticity, but we do not fully understand what it is like for them to live with it. To learn more, we talked to 10 people who had arm spasticity after a stroke. They had just finished a therapy programme to help with their arm. They told us how spasticity changed the way they felt about their body, their daily routines, and how they used health‐ and home‐care services. They also said they had to keep adjusting and finding new ways to cope with life after the stroke. The strategies they used helped them stay active and do daily tasks. Their experience may be different from other stroke survivors who have not had therapy. But their strategies can still help occupational therapists support others with arm spasticity after stroke.

**Key Points for Occupational Therapy**

- Stroke survivors with upper limb spasticity maintained or enhanced occupational participation by using self‐management strategies to adapt and adjust to activity demands in everyday life.
- Stroke survivors developed strategies, applying and extending skills and knowledge from rehabilitation experiences which included expecting, learning, practising, evaluating, and moving forward.
- Therapists working from a person‐centred enablement perspective can use these findings to explore the feasibility and utility of self‐management approaches with chronic stroke survivors who have upper limb spasticity.

## 1 INTRODUCTION

Post‐stroke spasticity is a common motor disorder characterised by involuntary muscular hyperactivity brought on by central neuronal lesions from the stroke (Dressler et al., 2018). Recent estimates derived from systematic review indicate spasticity prevalence of around 25% in all strokes and 40% in first time strokes with paresis (Zeng et al., 2021). People with spasticity after stroke have difficulty using their limb in everyday activities. They can also have problems with limb positioning and comfort, and in maintaining optimal condition of the limb. Cumulatively, spasticity can lead to adverse impacts on daily life, the ability to work, quality of life, independence, and mood (Barnes et al., 2017; Patel et al., 2020).

Qualitative evidence may inform rehabilitation practice decisions by illuminating the lived‐experience perspective. Reviews and meta‐syntheses of qualitative data have investigated a range of topics from the perspectives of the stroke survivor. Yet despite this substantial body of evidence, to date no review has investigated qualitative studies specifically relating to stroke survivors who have spasticity or the experience of having spasticity after stroke. A limited number of individual studies have explored the stroke survivor experience of living with spasticity. Kerstens et al. (2020) enquired about the experienced consequences of chronic post‐stroke spasticity with a focus on physical impairments and activity limitations, and the experienced effects of botulinum toxin treatment and whether current spasticity management addresses survivor needs. Interviews revealed spasticity‐related impairments and activity limitations within categories of stiffness, posture, pain and other sensations, loss of motor control, fatigue and shame, fluctuations in spasticity related to botulinum toxin, and the need for professional support and feedback (Kerstens et al., 2020). A further study using qualitative methods with stroke survivors who have spasticity (Levy et al., 2021) explored their views regarding, and factors impacting, adherence to an intense neurorehabilitation programme involving exercise. They found adherence could be explained by enablers in motivation (automatic, reflective) and opportunity (social), while barriers to adherence were capability (physical) and motivation (reflective). Neither of these studies explored stroke survivors experience of living with/having spasticity.

This study aims to address this gap and complement the emerging evidence base about the experience of spasticity post‐stroke. It explores the perspectives of community dwelling stroke survivors about their lived experience of having upper limb post‐stroke spasticity. In doing so, it aims to generate evidence to support implementation of evidence‐based, person‐centred neurorehabilitation that is informed by the ‘insider’ perspectives of stroke survivors themselves.

## 2 METHODS

To explore the experience of stroke survivors who have upper limb spasticity, a descriptive phenomenological approach was taken (Tavakol & Sanars, 2025). This approach was adopted because it enabled exploration of individual, first‐hand experience of a specific phenomenon—in this case that of living with upper limb spasticity (Sinfield et al., 2023) Further, this approach permitted description of the phenomenon from the participant perspective, revealing how they perceived, understood, and made sense of it. First person accounts were elicited from stroke survivors who had upper limb spasticity via interviews which were recorded and transcribed. They had recently completed an intensive rehabilitation programme designed for stroke survivors with upper limb spasticity. First person accounts therefore refer to that experience, as well as experiences in their everyday lives. Thematic qualitative analysis of interview data was used to identify shared meanings within participant experiences by identifying patterns and themes in the data (Kiger & Varpio, 2020). The *JBI Critical Appraisal Checklist for Qualitative Research* (Joanna Briggs Institute, 2017) informed reporting in this article. Human research ethics committee approval for this study was obtained as part of a multi‐site clinical trial by the Alfred Health Human Research Ethics Committee (442/12) which was registered at www.ANZCTR.org.au (ANZCTR12615000616572). The following paragraphs present participants, procedures, and approaches.

### 2.1 Participants

Prospective participants were stroke survivors who had recently completed a 12‐week outpatient intensive rehabilitation programme specifically designed for people with upper limb spasticity (Lannin et al., 2018, 2020). The programme included post‐botulinum toxin injection, serial casting, and evidence‐based motor training, with participants encouraged and reminded to practice movements in their home environment. After experiencing this programme, we thought these participants would be able to provide valuable insights about what it was like to have upper limb spasticity. Further, they would be accustomed to therapists asking about upper limb spasticity and may therefore be more aware of what spasticity was and more able to share their lived experience. Rehabilitation programme participants inclusion criteria were: greater than 3 months post‐stroke, scheduled to receive a botulinum toxin‐A injection to muscle(s) that crossed the wrist, not currently receiving upper limb rehabilitation, and having good cognition measured as <5 errors on the Short Portable Mental Status Questionnaire (Pfeiffer, 1975). There were 67 participants in the experimental group of the rehabilitation programme, 12 of these were invited to participate in interviews and 10 were able to do so. They were selected by the research team to obtain some variety in demographic attributes (age, self‐reported gender, productivity roles, and time post‐stroke).

### 2.2 Data collection

Demographic and clinical data of each participant was extracted from the rehabilitation programme records with the consent of interview participants. This included age, self‐reported gender, living situation, time since stroke, spasticity severity, cognitive status, pre‐stroke hand dominance, and affected upper limb. Time since rehabilitation programme completion was identified from the records. Participant data were recorded in a table that summarised their characteristics and is presented in the Findings section of this article.

The participants were interviewed by (author‐initials inserted‐blind‐for‐review) an experienced qualitative researcher who was an occupational therapist with a bachelor qualification in occupational therapy and a Masters (research) qualification in public health. The interviewer had not been involved in the delivery of the rehabilitation programme. Interviews were held at sites convenient to the participant: the outpatient clinic (*n* = 5), by phone (*n* = 3), at the person's home (*n* = 1), and at the local community library (*n* = 1). One interview was held with each participant; interviews went for as long as participants wished (range 26 to 69 minutes, mean 33 minutes, SD 14.66; median 33.5 minutes). Fatigue was a possible factor in the brevity of some interviews.

### 2.3 Materials

A broad, semi‐structured interview guide (Supporting Information) was used by the interviewer to elicit post‐stroke experience. Questions were open‐ended around topics relevant to the upper limb rehabilitation programme and the aim of this study. The guide opened with welcoming remarks and then invited participants to share their experience of ‘what it is like having spasticity’. Follow‐up questions and probes covered the topics of when they perceived their spasticity had started; what they noticed about spasticity as it developed; what they had performed previously to manage it; impacts on their roles, responsibilities, and ability to do things. The interviewer also used recursive questioning techniques whereby a topic or comment made by a participant would be used to prompt the next enquiry.

### 2.4 Data management

Interview audio recordings were transcribed by an independent person specifically employed for this task. Interview data were de‐identified prior to use by the research team and pseudonyms allocated. Demographic and clinical data for each participant was matched by the independent person to the pseudonym. Transcripts were uploaded into NVivo 12 (Lumivero, 2017) by SP.

### 2.5 Data analysis

This study aimed to describe participant experience of living with spasticity from their perspective. Demographic and clinical characteristics extracted from the rehabilitation programme record were tabulated and presented in results. Because there were only 10 people, there was no need to aggregate these data. Transcribed interview data were then explored using the six‐step method proposed by Braun and Clarke (2006, 2021) for thematic qualitative analysis. The worked example of thematic qualitative analysis provided by Kiger and Varpio (2020) was the procedural guide for this process.

Authors SP and AC were involved in Step 1 through Step 3 and Step 5, with other members of the research team involved in Step 4. In Step 1, two authors (SP, AC) familiarised themselves with the data through reading and re‐reading transcribed interview material, taking notes about issues, points of connection, and topics raised for further reflection in the next step of the process. The authors did this separately and then met to discuss perceptions we had about the data. In Step 2, initial codes were developed through inductive reasoning to try and capture the experience of stroke survivors who had upper limb spasticity. Codes included things like activities, sensations, places, people, and actions the participant identified in the interview. In Step 3 of the analysis, codes and the data extracts were examined to search for themes that helped make sense of experience presented by participants. Broad themes were identified around understandings of spasticity, the experience of the body in time and in a social and physical environment, service use, and specific actions or behaviours participants did which they thought helped them adapt or adjust. In Step 4, these emerging themes were reviewed by re‐reading all codes and data extracts and refining the number of themes and reflecting on relationships between themes. At this point in analysis, themes were identified, and the formulation presented in results was apparent. Authors SP and AC de‐identified‐for‐review presented the emerging findings to the research team for their reactions, input, and further refinement of the number and scope of themes. The Step 5 of thematic qualitative analysis involved revision of the final themes to name them and ensure they were internally consistent and did not overlap too much. Finally, themes and data extracts were then organised into tables for reporting and narrative explanation in this article. This is presented in the Findings section that follows. The participants were not consulted. To report findings, themes are presented in bold font, grouped into two overarching processes which are represented in *italics*.

## 3 POSITIONALITY STATEMENT

Authors one, two, three, five, and six are experienced occupational therapists with neurological rehabilitation expertise. Author four is an experienced rehabilitation physician. As rehabilitation professionals, we all have a desire for evidence that will inform action to enable recovery. This may have influenced the way we approached the topic of what it is like to have spasticity, the questions asked in the interview guide, what we chose to report as data examples, and the way in which the data were understood. The findings reveal descriptions of participant experience, ways they made sense of having upper limb spasticity, ideas about the impact on everyday life, and strategies they had used or devised themselves to live with upper limb spasticity. It is possible the author commitment to active rehabilitation, and enablement may have emphasised the focus on strategies to adapt. Three of the authors (NAL, MP and ES) were involved in the rehabilitation programme that our study participants completed. They were not involved in thematic qualitative analysis until the end of Step 4 when the themes and codes were presented for team review and discussion. No substantial changes were made. Overall, researcher positionality was orientated towards finding out information that would be useful in understanding and supporting recovery from a survivor perspective.

## 4 FINDINGS

Of 12 participants invited, 10 agreed to participate (nine males: median 49 years). One participant had his spouse also participate in the interview. The participants were 5.9 (2.95) years post‐stroke at the day of interview. The pre‐stroke dominant upper limb was affected in four of the 10 participants; all had diagnosed spasticity ranging from slight (*n* = 1) to severe (*n* = 3) as measured by the Tardieu Scale (Patrick & Ada, 2006), and all had very low levels of arm and hand use in their affected upper limb as measured by the Box and Block Test (Platz et al., 2005). All participants had either mildly reduced or intact cognition, and the therapist researcher identified during interview that two experienced expressive aphasia (word finding and word‐order problems when speaking) but could communicate meanings and experience clearly.

### 4.1 Key findings

The participant data revealed that lived experience was framed by two over‐arching themes. First that lived experience is *contextualised* in the *body*, in *time*, in a *life situation* and in relation to *access to and use of health and home‐care services and technology*. Second, experience as a stroke survivor with post‐stroke spasticity starts with a stroke but never ends. It is *continuous adaptation and adjustment* using processes of *expecting, learning, practising, evaluating*, and moving forward.

### 4.2 Lived experience of upper limb spasticity post‐stroke is contextualised

Stroke survivors with upper limb spasticity *contextualised* their experience in the *body*, in *time*, in a specific *life situation*, and in relation to *services and assistive technology access and usage*.

#### 4.2.1 The body

The participants contextualised their experience of spasticity within an individual body part or body parts. They described how the spasticity felt, their ability to move and control movement, their lack of movement, their sensation, and strength, specifically whether they were the same, reduced or increasing. The participants also discussed how their experience of spasticity had or had not changed over time.

*‘I think it's* [upper limb spasticity] *the same, mild, or a little bit actually decreased” (Joh); “it only moves – that's what I say – it only moves when I yawn because I've got a reflex action’* (Che)

The participants varied in the language they used when describing the body part affected by spasticity, suggesting they considered the upper limb to be part of them.

*‘… when I had to move my arm, well I'd use my whole shoulder, because both my mind and my hand didn't work properly’* (Bob)

While others used language such as ‘the’ or ‘it’ when referring to the affected upper limb, suggesting it was external or no longer felt part of them. One participant personified her upper limb affected by spasticity.

*‘that's why I have Trevor* [the name given to the upper limb with spasticity] *and my leg I have an AFO* [ankle foot orthosis]*’* (Kim)

The participants experience of spasticity contextualised within the body was discussed by either using the word spasticity or describing the feeling of their spasticity particularly referring to when it was active or less active.

*‘The spasticity is a bit of a bugger some days and I'd get, not seizures as such, but sort of like cramping and that in the leg and a tremor in my left arm. That's a real bloody nuisance sometimes … when I've been sitting down I'll stand up and my arm starts to tremor. I have to grab it and get it under control so its normal’* (Tom)

#### 4.2.2 Time

The experience of living with upper limb spasticity was contextualised in time. The participants spoke of specific points in time, such as time of stroke and time post‐stroke. Specific dates, the number of years or months post‐stroke, or when they recalled first noticing the development of spasticity were discussed.

*‘It* [spasticity] *started that moment* [of the stroke] *I can remember the first week. My hand was paralysed but also shaking’* (Joh)

Other participants contextualised the experience within time as a point when there was a change in their life situation including relationship *‘We dated a month before the stroke’* (Joh) or housing changes: ‘*So I suggested that I buy the other half* [of daughter's property to live there and get her help] … [we] *we're just about to leave* [their previous home]’ (Bob).

#### 4.2.3 Life situation

The experience of upper limb spasticity was contextualised within life situations which comprised multiple roles that participants maintained, adopted, or lost. This included roles within a family and relationships including fiancé, spouse, divorcee, parent, and adult–child of parents; ‘[I've got] *not a lot of friends. I've got a mate who drops around every now and again and my youngest son pops in on a regular basis so I catch up with him’* (Tom); in productive roles, including employment pre‐ and post‐stroke, domestic task roles, and leisure roles both pre‐ and post‐stroke.

*‘I'm working almost full‐time as a disability support worker or a carer, and so that is similar to cleaning the house, caring for the clients, cooking. I'm pretty good at that’* (Joh)

*‘I was going fishing, I knew I couldn't do what I used to, that means fly fishing. You need both hands to do it. Normal fishing that means you have to put the bait on the hook without squeezing it, and that's what I was doing because I couldn't control my left* [hand]. *I started slowly, slowly* [and] *I find out it* [hand] *works’* (Kit)

Life situations also included home living arrangements, where participants spoke of relocating after stroke because of separation and then divorce, relocation owing to the death of a partner and relocation to receive activities of daily living assistance from family, or staying in the same home with family visiting or partner moving in to provide assistance.

‘[after the stroke] *I separated with my wife and my three kids are still in* [City name]*’* *(Zac)* .

*‘I was living alone, yeah. I was finishing high school* [but came home for help from parents for] *general stuff like showering’* (Dan)

#### 4.2.4 Services and assistive technology access and usage

The participant experience was contextualised in relation to services and assistive technology access and use. The participants discussed health services that they utilised since their stroke including spasticity clinics, outpatient services. and telerehabilitation services. The participants also spoke of the individual health professionals that they had accessed, including occupational therapists, physiotherapists, neurologists, and unspecified health professionals.

Home and community care services were accessed by most participants and included government subsidised in‐home domestic chores assistance and community participation assistance.

*‘I have a home help sort of person and I can use them up to nine hours a week and I decide what we're going to do … usually we go for coffee or something like that because at the moment I feel a little bit stuck in my four walls so to speak’* (Kim)

*I have a PCA* [personal care assistant] *come once a week and takes me shopping and paying bills and that. Then she'll come back and cleans for me. She does pretty much the things I can't do like change the sheets on my bed and makes it, vacuums for me and mop floors and clean the bathroom’* (Tom)

Finally, assistive technology use was discussed including mobility technology, electrical stimulation technology, prescribed orthoses, and do‐it‐yourself devices (like the paint ball roller below) to help with positioning.

*‘I've got a little cart I sometimes use* [to go to shops], *I zoom off around there sometimes’* (Bob)

*‘Well of a night I have a paint roll holder or whatever, and I sleep with it of a night so that* [the hand fist] *it's out … It sort of, for me, to get my palm out’* (Kim)

### 4.3 Lived experience of upper limb spasticity involves continuous adaptation and adjustment

Living with post‐stroke spasticity involved *processes* of adaptation and adjustment implemented in the body, time, life situation, and services/assistive technology contexts previously described. Zac says what this is like: ‘*I haven't been able to use my left arm at all, which has been difficult. So, everything I've been doing, I've had to re‐adjust. I do things differently these days. I have certain clamps and procedures that I do things with and I've had to adjust’*.

The adaptation and adjustment processes used by participants are *expecting*, *learning, practising*, *evaluating*, and *moving forward*.

#### 4.3.1 Expecting

The participants discussed processes of expecting. This process was composed of concepts of expecting no recovery, some recovery, and through to recovery of movement.

*‘I didn't expect much because I know that now that it is a slow process’* (Kit)

*‘I was just hoping something would happen. I* [had] *tried to get physio as often as I can because I thought well something might work sooner or later’* (Bob)

*I was expecting to get movement back in my hand, arm’* (Zac)

Secondly, the participants discussed expecting recovery of hand function on a continuum of holding or grasping an object through to recovery of hand function in general.

*‘… if the fingers would just grasp something so he could use the hand even 10 per cent or 20 per cent* (Lee and Val)

‘*I was hoping that I could be able to carry something in the hand’* (Tom)

#### 4.3.2 Learning

The participants described learning about stroke before experiencing the stroke, learning after stroke about stroke sequelae and about stroke rehabilitation. Participating in stroke rehabilitation was also conceived of as learning process.

*I thought oh, this couldn't be a stroke ‐ because I know about strokes from – in teaching* (Kim)

*‘I started to understand much better what I was supposed to do. I found then the exercise, well seemed to be really good, real better … I was doing it properly’* (Bob)

#### 4.3.3 Practicing

Practising involved participants practising rehabilitation upper limb exercises regularly at home and deciding when to stop practice, practising fitness via walking and gym exercises, and practising spasticity‐affected upper limb muscle stretches.

‘*I really push myself on my walking side of things, you know to walk as far as I can and that makes me feel better’* (Tom)

*‘Pretty much going to the gym five days a week. I'm not an amateur gym goer but I have a specific program with strength, power lifting, gymnastics’* (Joh)

#### 4.3.4 Evaluating

Evaluating comprised self‐evaluation of activities participants cannot do now post‐stroke such as holding objects, completing self‐care and domestic chores, employment, driving and, leisure.

*‘I used to work before that* [stroke] *I used to work as a baggage handler at the airport. Obviously when I had the stroke I had to stop that, I couldn't work. I couldn't drive, I couldn't get around’* (Zac)

*‘Then when I had my stroke, it was like I couldn't be bothered thinking about it* [preparing family Christmas lunch], *what to do and anything’* (Kim)

Evaluation also comprised activities participants can do now including completing self‐care and domestic chores, employment, driving, leisure, walking, and helping.

*‘I can do almost every normal activity on my own’* (Joh)

*‘I do all my own washing I do all my own cooking and wash my dishes and everything* … *I go shopping and I walk up and down every aisle of the supermarket …’* (Tom)

Evaluating also involved judgements about the severity of impairments including the presence of spasticity over time, the resting position of the upper limb, strength, and upper limb sensation over time. Further, judgements about sense of self and how participants are the same or how they are now different.

*‘I accept the fact now that I am the way I am and that I'm not going to get any better’* (Tom)

*‘I'm the same man. My personality didn't change, my life has definitely changed … but I I'm still, I think happy, funny. It takes me five minutes* versus *a one minute normal person, but I take my time* [changing bed linen]’ (Joh)

The participants evaluated various aspects of rehabilitation, including the perceived impact and worth of doing rehabilitation, the worth of exercises and stretches, and the worth of botulinum toxin‐A injections.

*‘I think that everything helps. I think this is a step in my progress’* (Zac)

*‘I think it's* [stretches] *maintaining and … not to increase the motion, but just to combat the spasticity because if not it will get like a claw and that will be a burden’* (Joh)

#### 4.3.5 Moving forward

The participants described their experience in ways that communicated a sense of moving forward in life. This comprised the experience of thinking about oneself and one's life then back when the stroke occurred,

*‘In the hospital I couldn't move it’* (Zac)

‘*When I went* [shopping] *to put my right hand out … I couldn't, I could only put my left hand out’* (Kim)

*now* when changes or lack of changes can be observed,

*‘Now I can do something and that's good. I keep trying something else’* (Bob)

and in the *future*, thinking about what they hoped will be.

*‘There's going to be no miracle cure and the best thing I can do is improve my stamina and my fitness, which I do now’* (Tom)

## 5 DISCUSSION

This study addresses a gap in research evidence, focussing on the experience of stroke survivors living with upper limb spasticity. Our participants' experience was contextualised in their body, in time, and in their life situation. We used the term ‘life situation’ to capture their descriptions of roles, circumstances, living arrangements, social, and physical environments. They shared specific insights into their experience of upper‐limb post‐stroke spasticity in these contexts and strategies they used in everyday life. Findings suggest that the experience of life with upper limb spasticity following stroke was a dynamic one for our participants. Multiple processes were used by individuals to adapt and adjust to post‐stroke life; a life with an upper limb that feels and looks different and can do much less than before because of spasticity.

Our findings reveal similarities and differences to prior stroke participant‐perspective research. There is similar content in the sharing of their recovery journey from the time of the stroke and inpatient treatment (Hafsteinsdόttir & Grypdonck, 1997) to present life where biopsychosocial consequences of stroke required continuous adaptation and adjustment (Lawrence, 2010; Liang et al., 2020; Lou et al., 2017; Murray et al., 2003; Salter et al., 2008; Sarre et al., 2014; Wray & Clarke, 2017). In addition, similar to previous qualitative research about stroke survivor experience, our participants identified stroke as a pivotal event that marked ‘time before’ and a ‘time after’ in their lives—a point of disruption and sudden change (Lou et al., 2017; Salter et al., 2008; Williams & Murray, 2013).

Our participants described disruption to and adjustment in their self‐identity (Satink et al., 2013), making declarative statements about their personal attributes and/or relating how they engage in occupations that describe themselves (Williams & Murray, 2013). They also described experience relating to disruptions and ways back to valued occupations, occupational identity, and roles (Martin‐Saez & James, 2021). Like previous research on post‐stroke life, the participants described never‐ending adaptation as a response to the stroke event and consequences (Salter et al., 2008; Sarre et al., 2014). These descriptions and adaptations were contextualised in unique life situations which captured many environmental factors previously found to influence opportunities for resumption of valued occupations (Jellema et al., 2017).

As participants in an upper limb rehabilitation programme for people with spasticity, it is not surprising that they talked about upper limb recovery and their past and present access to and experience of rehabilitation adding to existing qualitative evidence on these topics (Luker et al., 2015; Meads et al., 2020; Peoples et al., 2011). The range and specificity of adaptation and adjustment strategies used by our participants was instructive, adding to evidence regarding post‐stroke self‐management approaches (Pearce et al., 2015). Their recent rehabilitation programme asked participants to practice exercises at home, and ‘practising’ was one of the processes used by them. But other processes such as ‘evaluating’ and ‘learning’, they perceived they had devised themselves to adapt and respond to their unique embodied and contextualised experience of upper limb spasticity and life with stroke. These ‘survivor‐generated’ strategies may be valuable examples to share with other people in person‐centred stroke services that aim to empower participants (Luker et al., 2015; Peoples et al., 2011). The ‘survivor generated’ processes may be useful examples of self‐management because they are presented in first‐person accounts from a lived experience perspective. They contribute to evidence about ways to understand, characterise, and determine the effectiveness of post‐stroke self‐management (Fryer et al., 2016; Jones & Riazi, 2011). Further, as ‘survivor‐generated’ strategies, they provide evidence to that supports co‐creation approaches to stroke‐survivor rehabilitation (Dobe et al., 2023).

The participants identified that services and assistive technology were an important part of their experience, specifically in relation to access and use. From these first person accounts, it appears enabling access to and use of services, and assistive technology was an integral part of the ecology of the stroke survivors' life and was empowering, because self‐management strategies could then follow. The participants described making decisions about what and how to use assistive technology and services, in the context of their everyday life and their preferred self‐management strategies. This finding suggests that assessment and prescription of assistive technology or referral to and use of services may be a way to help empower stroke survivors to think about these resources from a self‐management perspective.

While impairment and activity limitations were described by our participants, the contexts and processes they shared focussed more on activity performance and participation. Our findings about contextualised experience and survivor‐generated strategies add to existing qualitative evidence about social participation and reintegration after stroke (Della Vecchia et al., 2021; Walsh et al., 2015; Zhang et al., 2022). Findings of this study also add to research evidence about adjustment, the implied interaction between the embodied stroke survivor, their context, and the things they need and want to do. The presence and prominence of such a serious physical impairment as upper limb spasticity highlights the interactive nature of these elements in the post‐stroke experience.

There is no doubt that the recent involvement of participants in a rehabilitation programme after botulinum toxin A made this sample unique. Their experience will therefore be different to survivors who had no access to such programmes. But interestingly, despite the rehabilitation programmes' focus on impairment reduction, the participants focussed their description of the experience of ‘having spasticity’ as one where contexts and processes were focussed around maintaining or enhancing activity and participation in everyday life. A methodological limitation of the study was the lack of consumer involvement and the brevity of some interviews which reduced our ability to construct a rich and contextualised description of experience for all participants.

## 6 CONCLUSIONS

This study revealed these stroke survivors with upper limb spasticity have adjustment and adaptation experiences similar to other stroke survivors. Uniquely, they describe their experience as one contextualised in the body, in time, and in their life situations. They try to stay active and engaged in valued everyday activities. They build on their experiences in rehabilitation, access home, and health services as much as they can and develop unique strategies to self‐manage upper limb spasticity to reduce impairment and engage in activity. These strategies are the novel contribution of this study. The description of survivor‐generated self‐management strategies for life with post‐stroke spasticity may be relevant to stakeholders who are interested in enabling self‐management from a survivor perspective.

## AUTHOR CONTRIBUTIONS

SP, AC and NAL led the research project, LC and ES collected the data, SP and AC led the data analysis and drafting of the manuscript. All authors contributed to the manuscript. All authors read and approved the final manuscript.

## CONFLICT OF INTEREST STATEMENT

The authors have no conflict of interest to declare.

## Supporting information


**Data S1.** Semi‐structured interview guide.

## Data Availability

The data that support the findings of this study are available from the corresponding author upon reasonable request.
