# Supplementary material for: What stroke survivors say about living with upper limb spasticity and how they manage it
Source: Aust Occup Ther J. 2025 Sep 4;72(5):e70045. doi: 10.1111/1440-1630.70045 (PMC12409601; doi:10.1111/1440-1630.70045)
Supplement: Supplementary file 1 — Data S1. Semi‐structured interview guide. [file AOT-72-0-s001.docx]

**Semi-structured interview guide**

| *Information about this study* | I would like to understand your views on the burden of spasticity after stroke - on you as an individual, on your family and friends, perhaps too, on the broader community and health system. |
| --- | --- |
| *About the stroke* | To start, do you mind telling me a little about your stroke? |
| *About spasticity* | And when did the spasticity start? How long have you had spasticity?  What did you notice as the spasticity developed? |
| *About support & therapy* | What type of support do you get from family, community health or others with looking after your health? |
|  | - - What kind of roles/responsibilities do you have in your family |
|  | - - Has having a stroke changed your role in the family? What about the spasticity- did that affect things, or were things pretty much the same? |
|  | - - Have you been able to do things since having the therapy that you had to stop doing since the stroke? |
| *Final thoughts* | Do you have any final thoughts / Is there something else that you would like to say, that we have not talked about in this interview? |
